# LINC01235-TWIST2 feedback loop facilitates epithelial–mesenchymal transition in gastric cancer by inhibiting THBS2

**DOI:** 10.18632/aging.103979

**Published:** 2020-11-18

**Authors:** Yu-En Tan, Yao Xing, Ban-Lai Ran, Chao Zhang, Si-Wei Pan, Wen An, Qing-Chuan Chen, Hui-Mian Xu

**Affiliations:** 1 Department of Surgical Oncology, First Affiliated Hospital of China Medical University, Shenyang, China; 2 Department of Cell Biology, Key Laboratory of Cell Biology of Ministry of Public Health, and Key Laboratory of Medical Cell Biology of Ministry of Education, China Medical University, Shenyang, Liaoning, China

**Keywords:** gastric cancer, WGCNA, LINC01235-TWIST2-THBS2 axis, EMT

## Abstract

Although the anomalous expression of long non-coding RNAs (lncRNAs) has been extensively investigated in numerous carcinomas including gastric cancer (GC), their function remains unclear. The aim of our study was to explore the role of LINC01235 in GC. We used real-time quantitative PCR (RT-qPCR) to measure the expression of LINC01235 and twist family bHLH transcription factor 2 (TWIST2) in GC tissues. Scratch and transwell assays were performed to evaluate cellular capacity for migration and invasion. Gene relationships were explored by Weighted Gene Co-Expression Network Analysis (WGCNA). We measured TWIST2, thrombospondin 2 (THBS2) and epithelial-mesenchymal transition (EMT)-related proteins with western blot. We also used Pearson correlation analysis and the Kaplan–Meier method to detect associations among genes and overall survival. We found that LINC01235 was upregulated in GC tissues and cells. LINC01235 down-regulation restricted migration and invasion. Interestingly, we found the LINC01235-TWIST2-THBS2 axis induced EMT. Additionally, TWIST2 upregulated LINC01235 transcription in luciferase and chromatin immunoprecipitation (ChIP) assays. Bioinformatics analysis showed that microRNA (miR)-6852-5p might be a key gene involved in the regulation of TWIST2 by LINC01235. The LINC01235-TWIST2 positive feedback loop mainly affected migration and invasion of GC cells, which suggests it may serve as a potential therapeutic target in gastric cancer.

## INTRODUCTION

Gastric cancer (GC) is the fifth most commonly diagnosed cancer and the third leading cause of cancer death [1, 2]. Although, improved therapeutic techniques and modalities have significantly increased long-term survival for patients with GC, those who are diagnosed with middle and late-stage disease still have high mortality [3, 4]. Understanding the mechanisms of invasion and metastasis in GC will be critical for developing satisfactory therapies for these patients.

Long-chain non-coding RNA (long non-coding RNA, lncRNA) are RNA molecules longer than 200 nucleotides that do not encode protein but do affect various cellular processes [5, 6]. LINC01235 is located on chromosome 9p23, and is expressed in urinary bladder, stomach and 12 other tissues. LncRNA expression is particular to specific tissues under specific circumstances, and varies among different tissues; it has characteristic expression patterns in tumors and other diseases [7, 8]. LncRNA affects epigenetic regulation as well as transcriptional and post-transcriptional regulation of cancers. Sun et al. [9] found that lncRNA GClnc1 can promote GC angiogenesis through histone modification. Zhou et al. [10] suggested that gastric cancer metastasis-associated long noncoding RNA (GMAN) promotes translation of liver protein A1 through competitive binding to antisense GMAN-RNA, which greatly affects metastasis in GC.

Weighted Gene Co-Expression Network Analysis (WGCNA) is an efficient and accurate data mining method, dedicated to finding co-expressing gene modules and exploring relationships between gene networks and phenotypes proposed by researchers [11, 12]. Modules are groups of genes with similar expression profiles. If certain genes always change expression in similar ways during a physiological process, even in different tissues, these genes can reasonably be assumed to be functionally related. Co-analysis of mRNA and lncRNA together, as a module, generates information for both RNA types. As few lncRNAs have known functions, this strategy has been very helpful in finding lncRNAs that are closely related to better-known mRNAs, thus effectively narrowing the ranges of candidate lncRNAs and offering clues to their functions [13, 14].

In this study, we analyzed lncRNA expression and prognosis using The Cancer Genome Atlas (TCGA) database and explored the role of a particular lncRNA, LINC01235, in GC. Our paper reports for the first time, to our knowledge, that LINC01235 exerts a tumor promoting effect in GC. Mechanistically, we found when LINC01235 was knocked down in GC cells, twist family bHLH transcription factor 2 (TWIST2) activity was also repressed, thereby suppressing the EMT signaling pathway. MicoRNA (miR)-6852-5p appears to play an important sponging role in this process. We also found that overexpression of LINC01235 results in up-regulation of TWIST2 and downregulation of thrombospondin 2 (THBS2) activity in GC. In recovery experiments, an exogenous THBS2 plasmid reversed the effect of TWIST2 on EMT. More importantly, TWIST2 in turn upregulated LINC01235 expression in turn.

## RESULTS

### Identification of LINC01235 as a cancer-promoting lncRNA in GC

**Figure 1:**
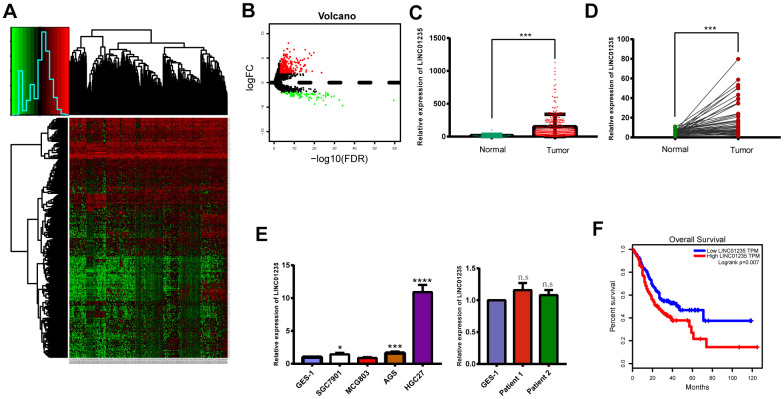
**LINC01235 is significantly upregulated in GC tissues and cell lines.** (**A**) Heatmap of TCGA-counts data. (**B**) Volcano plot of significance of gene expression. The x-axis shows significance by the -log10 transformed p-value number and the y-axis shows the gene expression difference. A gene is considered significantly differentially expressed if its |log (FC)| > 2 and the p-value < 0.05. Red represents the up-regulated genes while green represents the down-regulated genes. LINC01235 expression was compared between (**C**) GC tissues and adjacent normal tissues from TCGA, (**D**) 48 pairs of GC tissues and adjacent normal tissues, and (**E**) normal cell lines, GC cell lines and normal tissues. (**F**) Overall survival of GC patients from TCGA database. Data are presented as the mean ± SD. *, ** and ***: P < 0.05, 0.01 and 0.001, respectively.

To assess lncRNA expression in GC tissues, we analyzed total transcriptome expression and survival profiles from the TCGA STAD data set (Figure 1A, 1B). We also screened 18 differentially expressed lncRNAs, including 12 upregulated and 6 downregulated (Table 1). Finally, we chose LINC01235 as our research focus. Using Student's t-tests and censored survival analysis of the microarrays, we found LINC01235 expression was significantly associated with overall survival and differed significantly between cancerous and non-cancerous tissues (Figure 1C, 1F). To verify these results, we compared LINC01235 expression in GC cell lines and non-cancerous tissues with that in normal human epithelial cells (GES-1), and in the 48 paired GC samples with their adjacent normal tissues (Figure 1D, 1E). We found LINC01235 levels were highest in HGC-27 cells, and differed significantly between the paired cancerous and non-cancerous samples. We therefore focused on LINC01235 for further study. Analysis of clinicopathological characteristics showed that LINC01235 was associated with histological type (P = 0.023) and T stage (P = 0.016; Table 2).

**Table 1:** Significance analysis of microarrays (SAM) using the TCGA gastric cancer data set identified lncRNAs associated with patients’ overall survival.

**LncRNAs**	**P-valve**
**High expression correlated with shorter OS**	
LINC00973	0.005
LINC02293	0.007
LINC02407	0.007
LINC00326	0.009
LINC01235	0.010
LINC00601	0.011
LINC01929	0.033
LINC01940	0.036
LINC01967	0.039
LINC01526	0.041
LINC02253	0.046
LINC01297	0.048
**Low expression correlated with shorter OS**	
LINC02310	0.025
LINC02269	0.029
LINC00052	0.030
LINC00582	0.037
LINC02268	0.037
LINC01497	0.046

**Table 2:** Correlation between LINC01235 expression and clinicopathologic characteristics in GC patients.

**Factor**	**LINC01235 expression**		**P value**
	**Low (n=24)**	**High (n=24)**	
Sex			0.525
Male	16	18	
Female	8	6	
Age			0.386
≤65	10	13	
≥65	14	11	
Tumor location			0.798
Upper	5	5	
Middle	8	4	
Lower	11	13	
Histological type			
Well/moderate	10	3	**0.023**
Poor	14	21	
T stage			**0.016**
T2	10	2	
T3	7	7	
T4	7	15	
N stage			0.931
N0	7	6	
N1	5	5	
N2	4	3	
N3	8	10	

### Silencing LINC01235 inhibits migration and invasion of GC cells *in vitro* and *in vivo*

**Figure 2:**
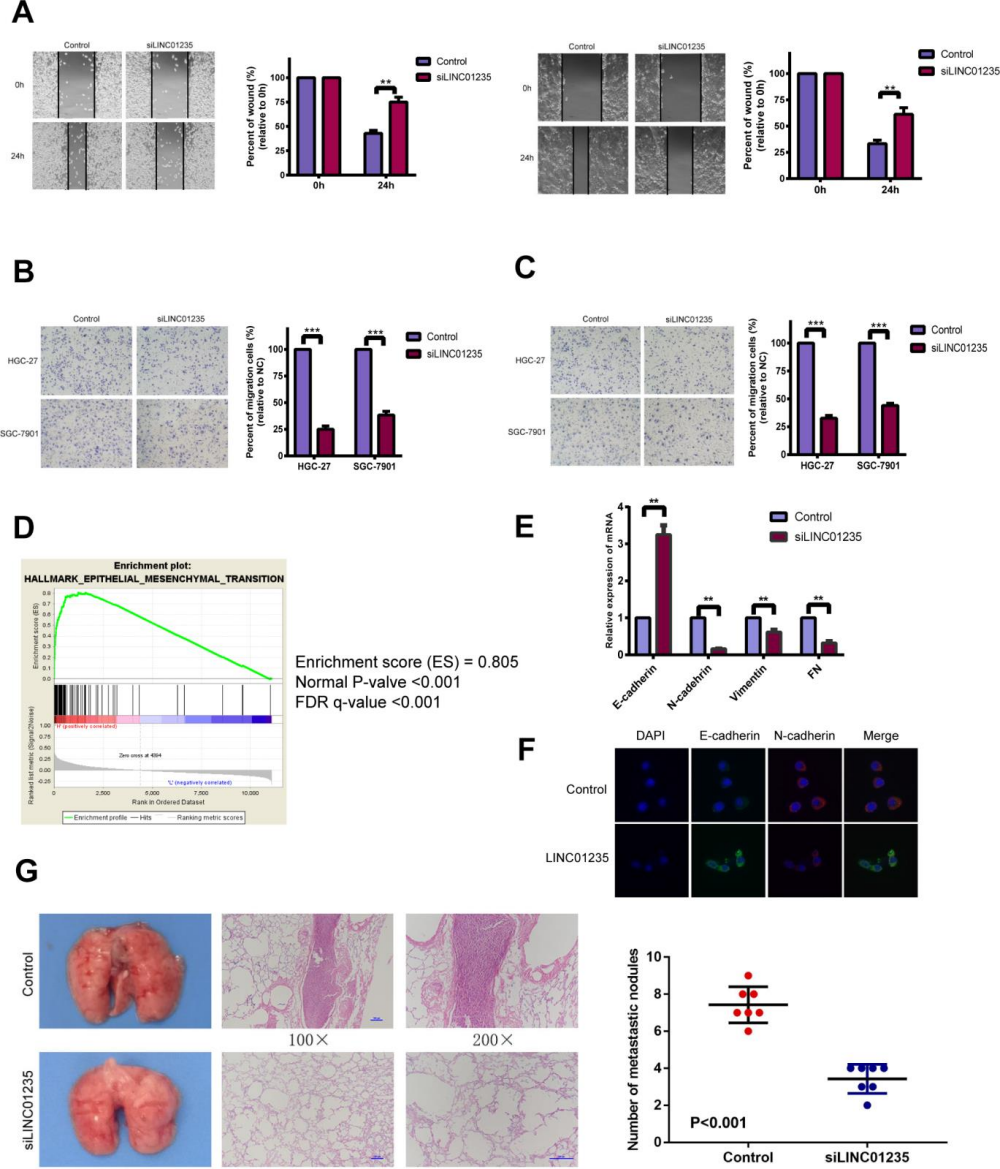
**LINC01235 affects gastric cancer metastasis *in vitro* and *vivo*.** (**A**) Wound-healing percentages at 24 h were largely inhibited in LINC01235-depleted HGC-27 and SGC-7901 cells, compared with control. In (**B**) migration and (**C**) Matrigel invasion assays, the number of migrated cells significantly decreased in LINC01235-silenced HGC-27 and SGC-7901 cells, compared with the control. (**D**) GSEA shows that LINC01235 may have a vital function in EMT. (**E**) RT-qPCR analyses of effects of LINC01235 depletion on expression of EMT-related genes in HGC-27 cells. (**F**) Immunofluorescence assay shows the expression of EMT in control/LINC01235-silenced cells. Green indicates E-cadherin and red indicates N-cadherin. (**G**) Representative tumor nodules in lungs of nude mice that were intravenously injected with control and LINC01235-silenced HGC-27 cells. Results are expressed as means ± SD. ** and ***: P < 0.01 and 0.001, respectively.

EMT is an essential process in metastasis, which affects GC occurrence and development. We chose HGC-27 and SGC-7901 cell lines for further study. Wound-healing assays showed that silencing LINC01235 markedly reduced GC cell mobility (Figure 2A). Transwell migration and invasion assays also showed that silencing LINC01235 inhibited GC cell migration (Figure 2B, 2C). Gene set enrichment analysis (GSEA) with data from TGGA STAD showed LINC01235 expression was positively related to EMT characteristics (Figure 2D).

To verify that LINC01235 regulates EMT, we examined mRNA levels of EMT-related genes, including E-cadherin, N-cadherin, vimentin and fibronectin(FN), in HGC-27 cells. We found that compared with the control group, the silenced group had a higher level of E-cadherin and a lower level of N-cadherin, vimentin and FN (Figure 2E). Immunofluorescence staining confirmed that the silenced group reversed the epithelial gene suppression and the upregulated expression of mesenchymal genes compared to the control group (Figure 2F). To further ascertain the effect of LINC01235 on GC metastasis *in vivo*, we then injected HGC-27/shLINC01235 or HGC-27/control cells into the tail veins of nude mice, and counted the number of their lung metastatic nodules at 8 weeks. Mice injected with HGC-27/shLINC01235 had fewer and smaller lung metastatic nodules than did mice in the HGC-27/control group (Figure 2G).

### Functional pathway enrichment analysis of LINC01235 by WGCNA

**Figure 3:**
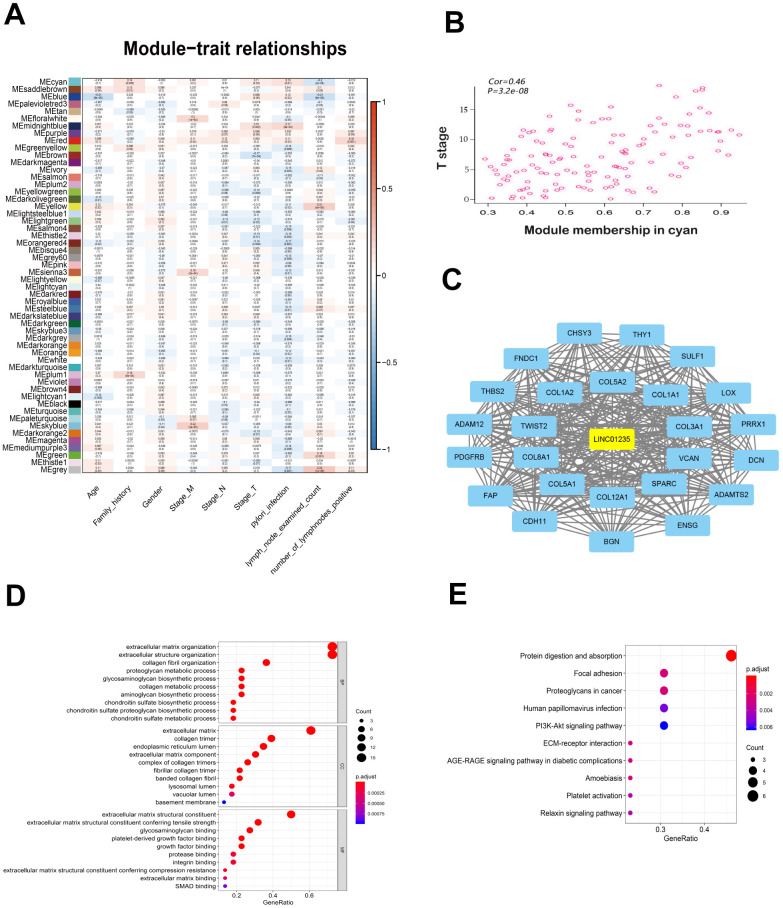
**WGCNA of LINC01235 with respect to signal modules.** (**A**) Correlation analysis between modules and clinical characteristics. Rows: module eigengene; columns clinical feature. (**B**) Scatter map of module members (cyan) and significance of primary tumor genes. (**C**) Network of modular gene relationships. GO (**D**) and KEGG (**E**) analyses of network relationships.

To identify potential LINC01235-associated genes, we used WGCNA to analyze gene expression in 375 patients with GC, and selected gene modules that were highly related to the patients’ clinical information to construct the network (Figure 3A). Eventually, LINC01235 was found to be highly related to T stage, in the cyan module (Figure 3B). We used Cytoscape 3.6.0 software to visualize network relationships in the cyan module (Figure 3C). GO analysis of the genes associated with LINC01235 showed that they were mainly involved in the composition of extracellular matrix (Figure 3D). KEGG analysis showed pathway enrichment in focal adhesion and ECM-receptor interaction (Figure 3E).

### LINC01235 regulates TWIST2 activity to influence EMT in GC

**Figure 4:**
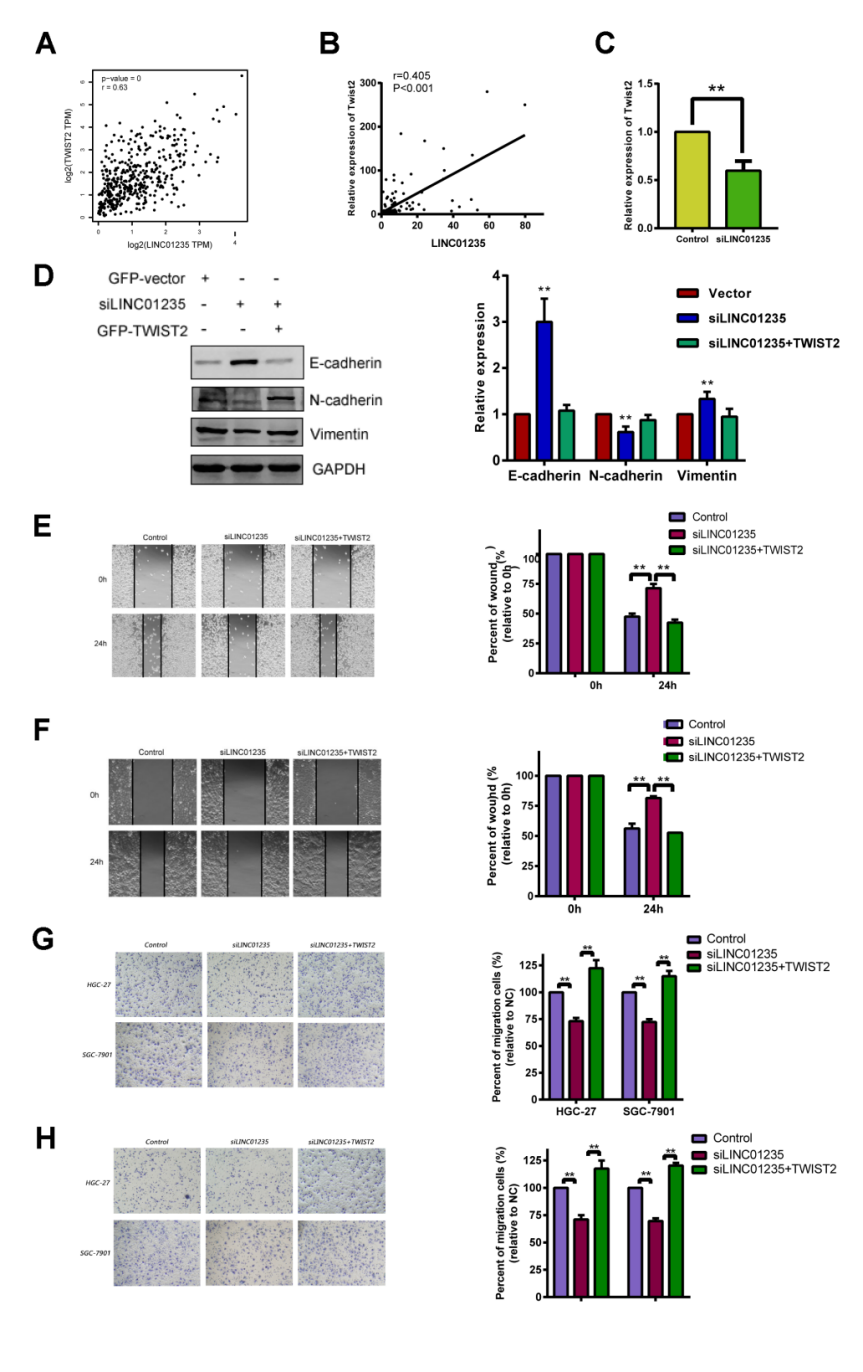
**LINC01235 depletion downregulates TWIST2 expression.** (**A**) Predicted correlation between LINC01235 and TWIST2 in the TCGA gastric cancer data set. (**B**) RT-qPCR shows correlations between LINC01235 and TWIST2 expression in 48 additional GC tissues. (**C**) RT-qPCR shows TWIST2 expression in control- and siLINC01235-HGC-27 cells. (**D**) Western blot shows effects of ectopic TWIST2 on expression of EMT-related genes in control- and siLINC01235-HGC-27 cells. (**E** and **F**) Wound-healing percentages in control, siLINC01235, and siLINC01235+TWIST2 groups. (**G** and **H**) Transwell assay of HGC-27 and SGC-7901 cells shows effects of LINC01235 and TWIST2 on migration and invasion. Results are expressed as means ± SD. **: P < 0.01.

Correlation analysis of TCGA showed that LINC01235 was positively correlated with expression of TWIST2 (Figure 4A). We then tested the 48 pairs of GC and noncancerous tissues, which showed similar results (Figure 4B). We used RT-qPCR to investigate the correlation between LINC01235 and TWIST2 expression in GC cells with knockdown of the LINC01235 gene; they showed significantly down-regulated TWIST2 expression (Figure 4C and Supplementary Figure 1). We next checked the effect of LINC01235 and TWIST2 on expression of EMT-related proteins. Western blotting showed that silencing LINC01235 sharply increased expression of E-cadherin and decreased the expression of vimentin and N-cadherin. However, overexpressing TWIST2 reversed mesenchymal gene suppression and downregulated expression of epithelial genes (Figure 4D). In the wound and transwell recovery assays, it was further confirmed that TWIST2 could reverse the migration and invasion ability of cells by silencing LINC01235 (Figure 4E–4H).

### THBS2 is a target of the LINC01235-TWIST2 axis in GC

**Figure 5:**
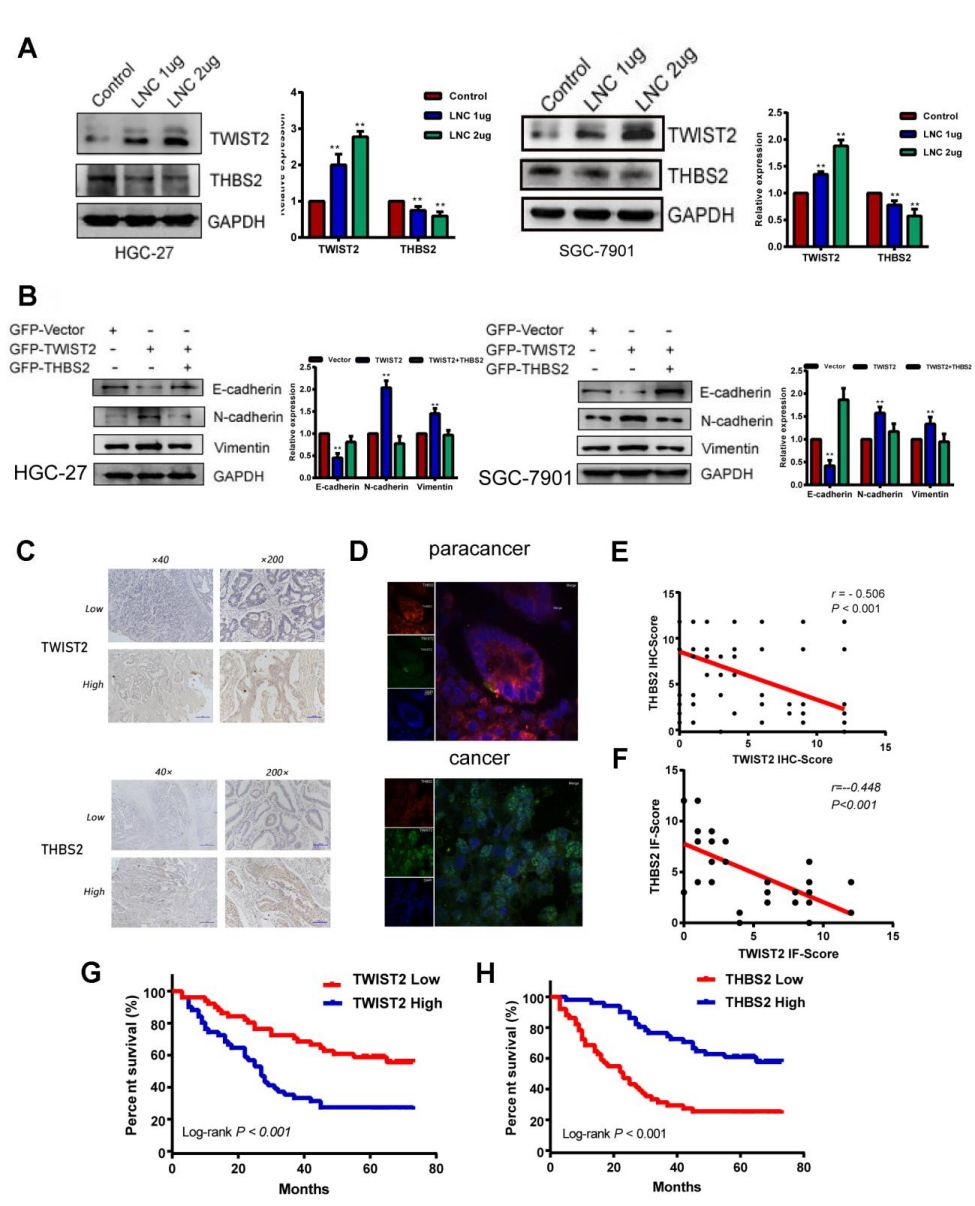
**THBS2 is the apparent target gene of the LINC01235–TWIST2 axis.** (**A**) The protein levels of TWIST2 and THBS2 were diminished by ectopic transfection of cells with strengthened LINC01235. (**B**) Western blot analysis of expression of E-cadherin, N-cadherin, and vimentin proteins in HGC-27 and SGC-7901 cells transfected with GFP-vector, GFP-TWIST2, and GFP-TWIST2/THBS2. (**C**) Low and high expression of TWIST2 and THBS2 in GC specimens. (**D**) TWIST2 and THBS2 expression by immunofluorescence staining in cancer and adjacent normal tissues. Green indicatesTWIST2, and red indicates THBS2. (**E** and **F**) Correlation between TWIST2 and THBS2 IHC scores and immunofluorescence scores. Kaplan–Meier analysis showed that (**G**) high expression of TWIST2 and (**H**) low expression of THBS2 predicted poor prognosis for GC patients.

TWIST2, is a transcription factor that modulates tumor invasion and migration through EMT, but its regulatory mechanism in GC is unclear. The THBS2 protein has been thought to inhibit EMT in gastric cancer [15]. As TWIST2 has been shown to be negatively correlated with THBS2 in cervical cancer [16], we transfected LINC01235 overexpression plasmids into HGC-27 and SGC-7901 cells (Supplementary Figure 2). Western blots indicated increased TWIST2 expression and a downward trend in THBS2 expression, which suggests that THBS2 may be a downstream target of the LINC01235–TWIST2 axis (Figure 5A). In the recovery experiment, THBS2 overexpression reversed the effect of TWIST2 on EMT (Figure 5B).

We next investigated TWIST2 and THBS2 expression in GC tissues from 102 patients by IHC. This analysis showed that TWIST2 and THBS2 positive expression rates were 52.0% (53/102) and 44.1% (45/102; Figure 5C). TWIST2 expression was negatively correlated with THBS2 expression (r = -0.506, P < 0.001; Figure 5E). Survival analysis associated high TWIST2 expression with worse prognosis, which was the opposite of THBS2 expression (Figure 5G, 5H). In immunofluorescence assays, we found that the intensity of green fluorescence (TWIST2) in cancer tissue was significantly higher than that of red fluorescence (THBS2), while in the adjacent tissues, we observed the opposite pattern (Figure 5D), and the difference of correlation analysis was statistically significant (r = -0.448, P < 0.001;Figure 5F).

### TWIST2 can regulate LINC01235 to form a positive feedback loop

**Figure 6:**
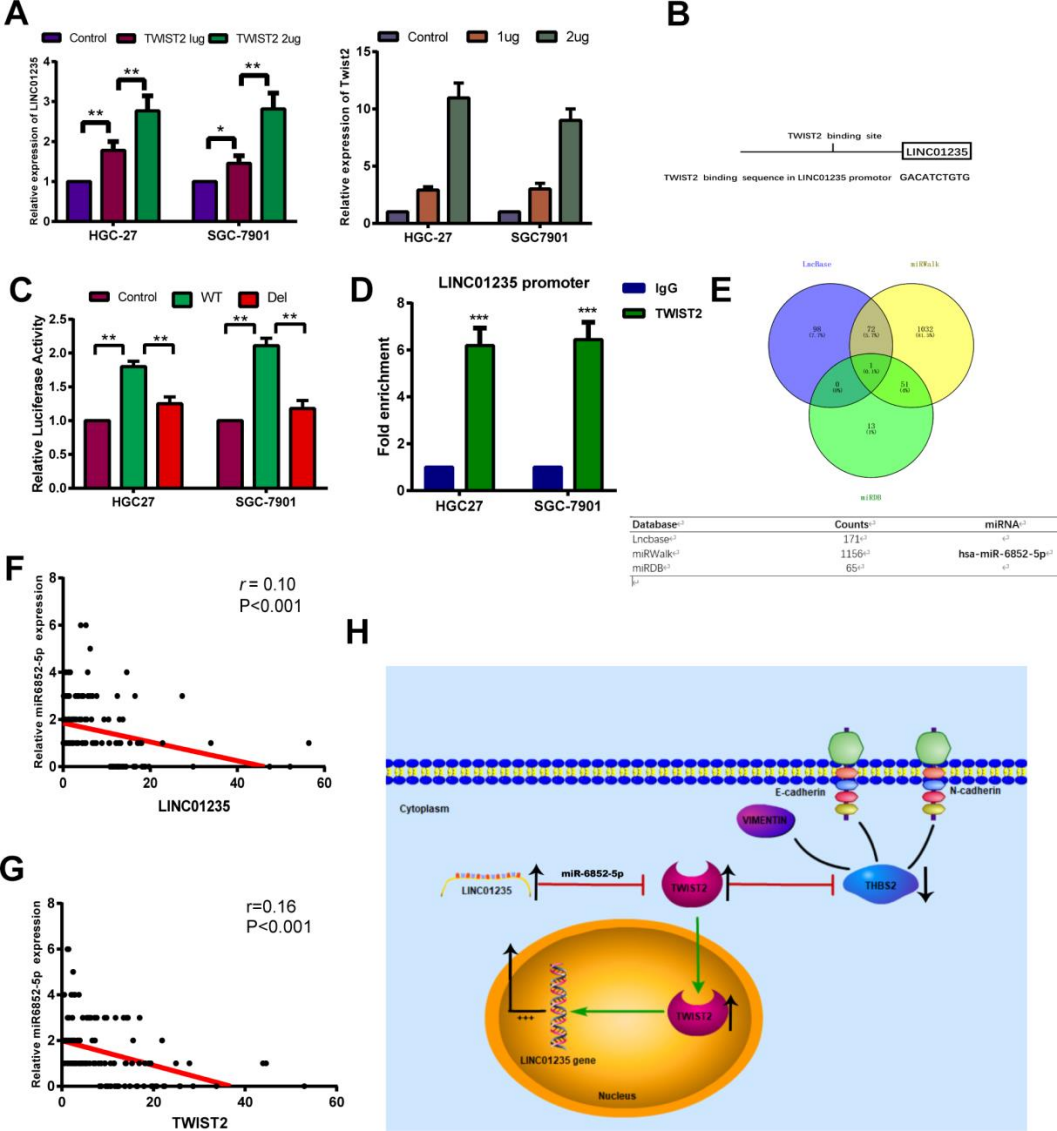
**TWIST2 activates LINC01235 expression through a positive feedback loop.** (**A**) RT-qPCR shows ectopic TWIST2 upregulated LINC01235 expression in HGC-27 and SGC-7901 cells. (**B**) Putative TWIST2-binding sites in the LINC01235 promoter. (**C**) Luciferase reporter assays confirmed TWIST2 activation of LINC01235 promoter through TWIST2-binding sites in HGC-27 and SGC-7901 cells. (**D**) ChIP assays were carried out using IgG and TWIST2 antibody in HGC-27 and SGC-7901 cells, followed by RT-qPCR with primers amplifying the LINC01235 promoter region. (**E**) Venn diagram indicates the miRNA acts as a sponge in three databases, including Lncbase, miRWalk and miRDB. (**F, G**) The correction of miR-6852-5p with LINC01235 and TWIST2 in TCGA database. (**H**) Schematic diagram shows the LINC01235–TWIST2–THBS2 axis; TWIST2, in turn, upregulates LINC01235. WT: wild type, and Del: deleted type. Results are expressed as means ± SD. **: P < 0.01.

Interestingly, the overexpression gradient of TWIST2 in HGC-27 and SGC-7901 cells showed that LINC01235 expression also increased (Figure 6A). We investigated the predicted binding site for TWIST2 in genomic LINC01235 at the Jaspar website (Figure 6B and Supplementary Table 2), and used luciferase testing to verify that ectopic TWIST2 expression enhanced transcription of firefly luciferase from the wild-type LINC01235 promoter. When the TWIST2-binding sequence was deleted, the firefly luciferase expression significantly decreased (P<0.01) (Figure 6C). We also performed ChIP assays to determine if TWIST2 bound to the LINC01235 promoter in cells. As expected, RT-qPCR results showed that the TWIST2 groups were higher than the IgG control groups (P<0.001; Figure 6D). In order to explore the regulatory effect of LINC01235 on TWIST2, we predicted and analyzed target genes in three databases, LncBase, miRWalk and miRDB. The Venn diagram shows that miR-6852-5p may be the key target miRNA involved in the regulation of TWIST2 by LINC01235 (Figure 6E). The TCGA database analysis showed that the expression of miR6852-5p was negatively correlated with LINC01235 and TWIST2, which further confirmed our idea (Figure 6F, 6G).

## DISCUSSION

Cancer metastasis is a critical hallmark of malignancy, with a complex and multi-step biological process [17, 18]. The initial stage of metastasis depends on an aggressive biological process, EMT, which is characterized by specific morphogenetic changes, including increased cell motility and loss of cell–cell adhesion. This study found a novel LINC01235–TWIST2–THBS2 signaling axis, which has an important function in GC metastasis (Figure 6H).

As possible factors in the mechanisms and biological processes of cancer, lnRNAs have been widely studied in recent years [19, 20]. Human genome analysis shows that LINC01235 is located on chromosome 9p23, and is expressed in urinary bladder, stomach and 12 other tissues. LINC01235 upregulation in breast cancer may represent a more aggressive phenotype [21]. However, the connection of LINC01235 with tumor invasion and migration has not yet been verified. We confirmed by RT-qPCR that LINC01235 was highly expressed in tumor tissues, and LINC01235 silencing inhibited EMT properties *in vitro*.

Constructing co-expression modules by the WGCNA algorithm to seek a potential key or prognostic significance gene has been widely used [22, 23]. In order to determine whether LINC01235 was associated with the clinical characteristics of GC, we performed the WGCNA analysis on RNA-seq data from TCGA gastric cancer patients. The division obtained 53 valid modules. The conjoint analysis between the gene expression data and the clinical pathological factors of the patients can estimate the relationship between the modules and the characteristics. The traits we selected included TNM stage, age, sex, family history, H. pylori infection, number of lymph nodes and number of positive lymph nodes. Then, the software was used for visualization. The key genes in the module can be determined based on the node-centered analysis. LINC01235 was enriched in the cyan module. Functional annotation analysis showed that LINC01235 was involved in the composition of extracellular matrix, focal adhesion and ECM receptor interaction.

LnRNAs have been shown to regulate downstream transcription factors [24–26]. When we chose LINC01235 as our research focus, we found its expression to be highly correlated with that of TWIST2. As a transcription factor, TWIST2 has been found to promote and indicate EMT in various cancers [16, 27, 28]. We found that expression of TWIST2 was reduced in LINC01235-deficient cells. Interestingly, overexpression of TWIST2 could increase LINC01235 expression in GC cells. We inferred that TWIST2 regulated LINC01235 transcription by binding to its promoter, which could ultimately form a positive feedback loop.

In summary, our results demonstrated that the LINC01235-–TWIST2–THBS axis promoted GC metastasis; this process was reversed when LINC01235 expression was repressed. These results imply that controlling LINC01235 levels may provide a novel GC therapy. Further research is warranted to prove the value of LINC01235 as a marker for diagnosis and outcome.

## MATERIALS AND METHODS

### Cell culture

We purchased one immortalized human gastric epithelial mucosa cell line (GES-1) and four GC cell lines (SGC-7901, HGC-27, MGC-803, and AGS) from the cell bank of the Cell Culture Collection of the Chinese Academy of Sciences (Shanghai, China). GES-1, SGC-7901, MGC-803 and AGS cells were cultured in DMEM medium supplemented with 10% fetal bovine serum (FBS; Biological Industries, Israel). HGC-27 cells were cultured in 1640 medium supplemented with 10% FBS.

### Samples and patients

We used 48 pairs of fresh GC specimens and adjacent non-cancerous tissue, from patients who underwent surgery in 2018 at the First Affiliated Hospital of China Medical University. We also examined 102 sets of paraffin-embedded GC tissues from patients treated between 2009 and 2012. All patients were pathologically confirmed to have gastric adenocarcinoma, with no tumors at other sites; none underwent preoperative radiotherapy or chemotherapy. The patients or their family members signed informed consent forms. This study was approved by our hospital’s Research Ethics Committee.

### TCGA analysis

We downloaded RNA-Seq data of stomach adenocarcinoma (STAD) patients from TCGA, and used WGCNA to build co-expression modules, and to investigate relationships between modules and clinical traits, in R language. Gene Ontology (GO) and Kyoto Encyclopedia of Genes and Genomes (KEGG) analyses were performed to assess the functional role of intersection genes [29, 30].

### Real time quantitative PCR (RT-qPCR) analysis

After extracting total cellular RNA, cDNA templates were generated by reverse transcription using a PrimeScript^TM^ RT kit (TaKaRa, Japan). RT-qPCR was performed to calculate relative expression of mRNA according to the reaction system. GAPDH was chosen as the reference gene, and U6 as an internal control for tissue samples. The primer sequences are shown in Supplementary Table 1.

### Vector construction and infection

LINC01235 - RNAi lentiviruses were constructed using GenePharma (Suzhou, China). HGC-27 and SGC-7901 cells (2 × 10^5^) in 12-well plates were infected with 20 μl lentiviral suspensions of 3 × 10^8^ TU/ml, using polybrene according to manufacturer instructions. Puromycin (Sigma, USA) at 5 μg/ml was used to select infected cells. Cells were transfected with GFP-LINC01235, GFP-TWIST2, GFP-THBS2 and their control plasmids (GeneChem, Shanghai, China), using Lipofectamine 3000 (Invitrogen, Beijing, China). The cells were harvested after 48h.

### Wound-healing assay

The cells were digested with pancreatin, the cell density was adjusted to 1×10^5^ cells/ml, and 2 ml of cell suspension was seeded in six-well plates. When the cell confluence reached 80%-100%, the plates were scratched with 200μl pipette tip. Floating cells were removed with PBS. FBS-free medium was then added, and the plates were photographed (100× magnification). The cells were then placed in an incubator at 37°C for 24 h, and the scratch was again observed under a microscope and photographed.

### Migration and invasion assay

The invasion chambers were rehydrated with Matrigel and DMEM (serum-free) for 2h at 37°C. Cells were resuspended in serum-free medium at 2 × 10^4^ cells/mL in 24-well plates. We added 600 μL of medium containing 10% FBS to Transwell chambers (8 μm pore size; Corning, USA), and 200 μL of cell suspension to the small chamber. cells were fixed and stained after 18 h, and cells that migrated were counted under the microscope, which was considered representative of migratory ability.

### Transcription factor-binding site analysis

Jaspar (http://jaspar.genereg.net), is an open database that helps predict potential transcription factor-binding sites in various species [31].

### Luciferase activity assay

The promoter region of LINC01235 was cloned into pGL3-promotor vectors (GENEWIZ, Suzhou, China). SGC-7901 and HGC-27 cells (5 × 10^5^) were seeded in 12-well plates and transfected with GFP-TWIST2 and reporter plasmids, using Lipofectamine 3000. After 48 h, the cells were collected using the dual luciferase reporter assay kit (Promega, USA) according to the manufacturer’s protocol. Renilla and firefly luciferase activities were measured by a fluorometer.

### Chromatin immunoprecipitation (ChIP) assay

Cells were incubated with 37% formaldehyde at 37°C for 10 minutes, then collected in lysis buffer with 600ul of 1% SDS. DNA was sheared by sonication to lengths between 200 and 1000 bp. ProteinDNA complexes were precipitated by antiTWIST2 antibody (Abcam, ab66031, USA) or control IgG, followed by elution of the complex from the antibody, using a ChIP kit (Millipore, 17-371, USA). RT-PCR was carried out with primers specific for the LINC01235 promoter region: 5′- ACTTTGTTATAGAATCTG - 3′ (sense) and 5′- GCTCTGAACTCATGTGAT - 3′ (antisense).

### Immunohistochemistry

Immunohistochemistry (IHC) staining was performed in accordance with standard protocols. IHC staining was assessed by scores based on the percentage of positive cells (0: < 5%, 1: 5%–25%, 2: 25%–50%, 3: 50%–75%, and 4: > 75%) multiplied by scores based on the intensity of staining, (0: colorless, 1: light yellow, 2: brown, 3: and dark brown), with scores of 6–12 considered high expression and 0–4 considered low expression. Primary antibodies used in IHC were purchased from the following sources TWIST2 (Proteintech, Chicago, USA) and THBS2 (ZENBIO, Chengdu, China).

### Immunofluorescence staining

We examined 15 paired cancer tissues and adjacent normal tissues. Tissues were fixed in 4% paraformaldehyde and then blocked with normal goat serum. Then, they were incubated with primary antibody for 1 h at 25 °C, washed with PBST containing 0.1% Triton X-100, and subsequently incubated with secondary antibody conjugated with green or red dye for 40min. DAPI was used for nuclear staining. The same experimental steps were used for the detection of EMT biomarkers in cells, using antibodies against E-cadherin (BD, 610181, USA), and N-cadherin (CST, 13116, USA). Confocal scanning analysis was performed using a Leika/Olympus laser confocal scanning microscope.

### Lung metastasis assay

For the xenograft model, 2 × 10^6^ HGC-27 cells were intravenously injected into the tail veins of 4-week-old female Balb/c nude mice, which were randomly divided into two groups with 7 mice in each group. Eight weeks after the injection, all mice were euthanatized, and their lung tissue was removed and embedded in paraffin for hematoxylin-eosin staining. All animal experiments were approved by the Animal Ethics Committee of China Medical University.

### Statistical analysis

We used SPSS 22.0 statistical software (IBM, USA) and GraphPad prism7.0 mapping software (GraphPad Software, USA). Student's t test was used to compare two groups. Data are presented as means ± standard deviation. The Kaplan–Meier method was used to calculate overall survival. Gene expression correlation was detected by Pearson’s analysis, and P < 0.05 was considered significant.

### Ethics approval

The experiment was approved by the Medical Ethics Research Association of the first affiliated Hospital of China Medical University, and each GC patient signed a written informed consent form. Animal experiments were carried out strictly in accordance with the nursing rules of experimental animals in the first affiliated Hospital of China Medical University.

## Supplementary Material

Supplementary Figures

Supplementary Tables
