# Supplementary material for: LINC01235-TWIST2 feedback loop facilitates epithelial–mesenchymal transition in gastric cancer by inhibiting THBS2
Source: Aging (Albany NY). 2020 Nov 18;12(24):25060–75. doi: 10.18632/aging.103979 (PMC7803553; doi:10.18632/aging.103979)
Supplement: Supplementary Figures [file aging-12-103979-s001.pdf]

SUPPLEMENTARY FIGURES

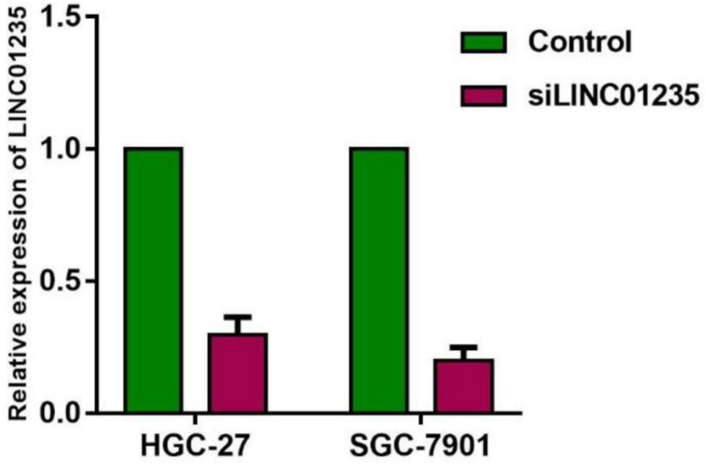

Supplementary Figure 1. LINC01235 silencing efficiency in HGC-27 and SGC-7901 cells.

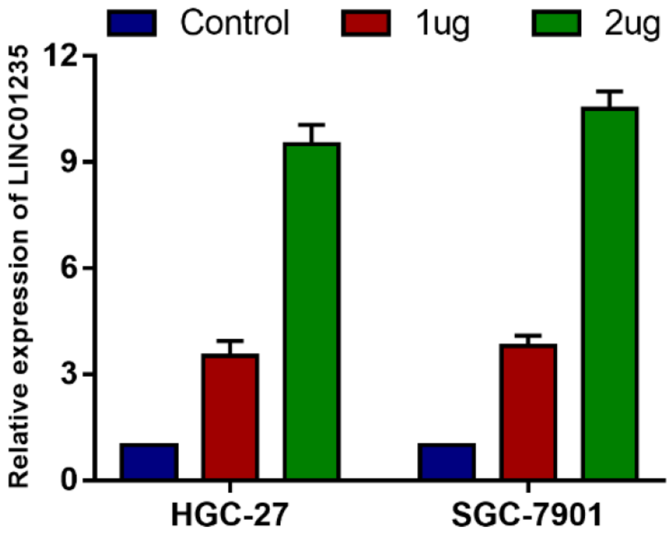

Supplementary Figure 2. LINC01235 overexpression efficiency in HGC-27 and SGC-7901 cells.
