# Supplementary material for: LINC01235-TWIST2 feedback loop facilitates epithelial–mesenchymal transition in gastric cancer by inhibiting THBS2
Source: Aging (Albany NY). 2020 Nov 18;12(24):25060–75. doi: 10.18632/aging.103979 (PMC7803553; doi:10.18632/aging.103979)
Supplement: Supplementary Tables [file aging-12-103979-s002.pdf]

SUPPLEMENTARY TABLES

Supplementary Table 1. The sequence of Forward primer and Reverse primer in qRT-PCR.

Gene	Forward primer	Reverse primer
LINC01235	CTCACAGGTCAACGCAGGAT	ACGTCAAGGCTCCTCAATCG
TWIST2	GCAAGATCCAGACGCTCAAGCT	ACACGGAGAAGGCGTAGCTGAG
THBS2	CAGTCTGAGCAAGTGTGACACC	TTGCAGAGACGGATGCGTGTGA
E-cadherin	GCCTCCTGAAAAGAGAGTGGAAG	TGGCAGTGTCTCTCCAAATCCG
N-cadherin	CCTCCAGAGTTTACTGCCATGAC	GTAGGATCTCCGCCACTGATTC
Vimentin	AGGCAAAGCAGGAGTCCACTGA	ATCTGGCGTTCCAGGGACTCAT
Fn	ACAACACCGAGGTGACTGAGAC	GGACACAACGATGCTTCCTGAG
GAPDH	GTCTCCTCTGACTTCAACAGCG	ACCACCCTGTTGCTGTAGCCAA
U6	CTCGCTTCGGCAGCACA	AACGCTTCACGAATTTGCGT

Supplementary Table 2. The predicted binding sequence of TWIST2 and LINC01235 in Jaspar website.

Predicted sequence	Score	Relative score	Start	End	Strand
GACATCTGTG	6.53	0.84	325	334	+
CACAAATGTG	5.99	0.83	1614	1623	+
CACATTTGTG	5.98	0.82	1614	1623	-
ATCACATGAG	5.78	0.82	408	417	+
CTCATGTGAT	5.57	0.82	408	417	-
CACAGATGTC	5.42	0.81	325	334	-
GGCACATGCC	5.19	0.81	741	750	+
GGCATGTGCC	5.14	0.81	741	750	-
